# Comparison of stress and strain distribution around splinted and non-splinted teeth with compromised periodontium: A three-dimensional finite element analysis

**DOI:** 10.15171/japid.2018.007

**Published:** 2018-06-20

**Authors:** Reza Amid, Mahdi Kadkhodazadeh, Farshad Dehnavi, Mahyar Brokhim

**Affiliations:** ^1^Dental Research Center, Research Institute of Dental Sciences, Dental School, Shahid Beheshti University of Medical Sciences, Tehran, Iran; ^2^Dental School, Shahid Beheshti University of Medical Sciences, Tehran, Iran; ^3^DDS, Private Practice, Tehran, Iran

**Keywords:** Finite element analysis, periodontal splinting, strain, stress

## Abstract

**Background:**

Splinting of teeth is performed for effective distribution of loads in mobile teeth and to lower the stress applied to compromised teeth. Biomechanics cannot adequately explain load distribution around natural teeth. This study aimed to compare the distribution pattern and magnitude of stress and strain around splinted and non-splinted teeth with compromised periodontium using three-dimensional (3D) finite element analysis (FEA).

**Methods:**

Six mandibular anterior teeth were scanned and data were registered in CATIA® and then SolidWorks® software programs. The jawbone was also designed. In the second model, the teeth were splinted with fiber-reinforced composite (FRC). The models were then transferred to ANSYS® software program and after meshing and fixing, 100- and 200-N loads were applied at zero and 30° angles. The magnitude and distribution of stress and strain in the periodontal ligament (PDL) and the surrounding cortical bone were determined.

**Results:**

A significant reduction in stress was noted in cortical bone around central and lateral incisors while an increase in stress was noted around the canine tooth after splinting. All these changes were more significant under 100-N load compared to 200-N load and greater differences were noted in response to the application of oblique loads compared to vertical loads.

**Conclusion:**

Splinting decreased the magnitude of stress and strain in teeth close to the center of splint and increased the stress and strain in teeth far from the center of splint. Adequate bone support of canine teeth must be ensured prior to selection of splinting as the treatment plan for the anterior mandible since it increases the longevity of all the teeth.

## Introduction

Treatment of teeth with advanced periodontal disease and severe mobility following bone loss includes a combination of periodontal treatment, occlusal adjustment, stabilization of mobile teeth and eventual extraction of hopeless teeth.^1^ Some techniques are used for management of mobile teeth such as guided tissue regeneration for teeth with vertical bone resorption defects,^2-4^ preparation and splinting of mobile teeth using conventional fixed partial dentures,^5^ tooth extraction and replacement with dental implants or splinting of teeth. In the latter technique, teeth with poor bone support are splinted and thus, tooth mobility is minimized.^6-8^ Different techniques are available for splinting of mobile teeth to their adjacent teeth;^9^ these techniques are divided into two groups of extra-coronal and intra-coronal techniques.^10^ Intra-coronal splints are not often recommended since they require tooth preparation and removal of tooth structure.^10,11^ The most commonly used method of splinting of teeth with periodontal mobility is the use of extra-coronal splints by use of composite resin along with adhesives, fiber-reinforced composite (FRC) or orthodontic wires along with composite resin.^9,11^ However, previous studies indicated that none of the three afore-mentioned techniques had any superiority to the others.^8,9,11-13^ Studies in this field have been limited. Thus, this study aimed to biomechanically assess two common treatment plans (with and without splinting) for teeth with compromised periodontium. Finite element analysis (FEA) is a quantitative method for stress analysis.^14^ This method solves complex problems by dividing them into smaller, simpler pieces. Several variables can be assessed as such. In this method, instead of finding a solution for the entire complex, a formulated solution for each finite element component is designed and solved and is then generalized to the entire complex.^8,14,15^ Several FEA studies have been carried out on dental implants and their comparison with natural teeth.^12,16,17^ However, comparative studies on periodontally compromised teeth have been scarce and adequate information is not available to reach a logical and reliable clinical decision.^18^ This study aimed to assess the magnitude and pattern of distribution of stress and strain around splinted and non-splinted teeth with compromised periodontium using FEA.

## Methods

This experimental study was carried out using FEA. SolidWorks (Version 2014) software program (SolidWorks Corp, Dassault Systèmes, USA) was used for final designing of models and ANSYS (Workbench 15; Dassault Systèmes, USA) was used for final analysis. First, two 3D models of the anterior mandible were designed. Model 1 had six mandibular anterior teeth in a resorbed alveolar ridge (7 mm of bone loss). Model 2 was the same as model 1 with the difference that the crowns of all the six teeth had been splinted to each other on the lingual aspect by FRC. To determine the accurate anatomical form of the teeth, first teeth with dimensions of natural teeth^15^ (Table 1) were carved out of wax blocks and then 3D scans were obtained of the wax patterns. Data were registered in CATIA software program (V5R2015X, Dassault Systèmes, France). After final corrections and ensuring correct form and anatomy of teeth (similar to those of natural teeth), data were registered in SolidWorks software program (Version 2014). To design the model of bone, first a CT scan was obtained of a dentate jaw and the mean dimensions of the mandible were used. Then, a model with 7 mm of horizontal alveolar bone resorption (over 50% of the mean length of roots) was designed which included both cortical and cancellous bone with natural thickness,^19^ a specific modulus of elasticity^20^ and a PDL space with a certain thickness and modulus of elasticity^21^ by the SolidWorks software program.

**Table 1:** Dimensions of mandibular anterior teeth designed in the dental model^15^

**Tooth**	**Labiolingual**	**Mesiodistal**	**Crown length**	**Root length**
**Central incisor**	6 mm	5 mm	9 mm	12.5 mm
**Lateral incisor**	6.5 mm	5.5 mm	9.5 mm	14 mm
**Canine**	7.5 mm	7 mm	11 mm	16 mm

Contact of teeth was defined as contact point as in normal anatomy by the SolidWorks software program. To design the second model, the teeth in model 1 were splinted together with FRC. The FRC band was defined to have 2×0.3-mm dimension with 38.5 GPa modulus of elasticity by the software program. It was attached to the teeth on the lingual surface right above the cingulum. After designing the models, they were transferred to ANSYS software program (Workbench 15, USA). Jaw bone in both models was fixed-supported 4 mm apical to the apex of canine in the inferior border of the mandible and 15 mm distal to the distal surface of canine tooth. Both models were meshed by ANSYS software program by fine mesh and the size of each element measured 0.3 mm (Table 2).

**Table 2:** Number of nodes and elements

**Models**	**Number of nodes**	**Number of elements**
**Non-splinted**	749608	501832
**Splinted**	723448	488338

**Figure 1:**
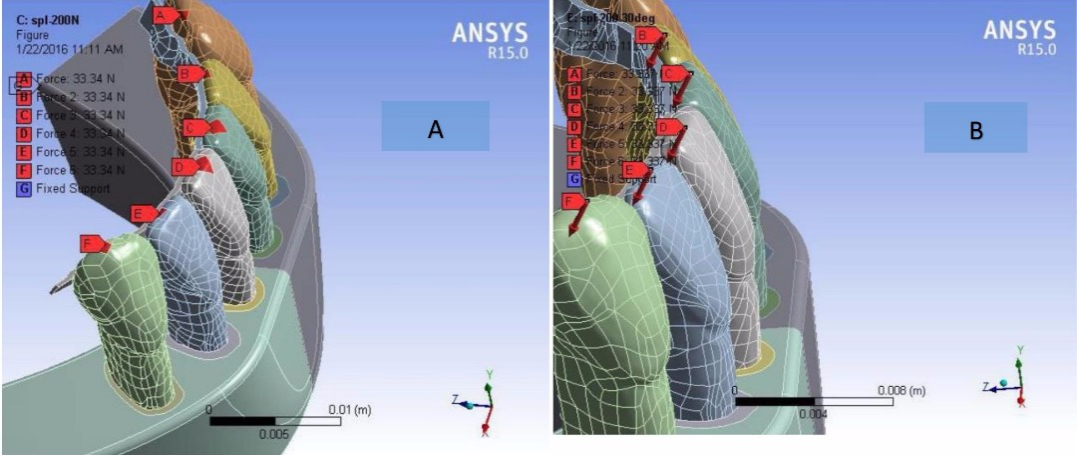
(A) Load application at 0° angle; (B) Load application at 30° angle.

Next, 100- and 200-N loads were separately applied to the models parallel to the long axis of the teeth (0° angle) and with 30° angle relative to this axis (four different positions) (Figure 1). ANSYS software program performed a comprehensive analysis of the magnitude and distribution of stress and strain following the application of these loads in the PDL and cortical and cancellous bones. The maximum von Mises strain, maximum shear stress, maximum von Mises stress and deformation were reported by the software.

## Results

### Maximum von Mises stress for the two splinted and non-splinted models

**Figure 2:**
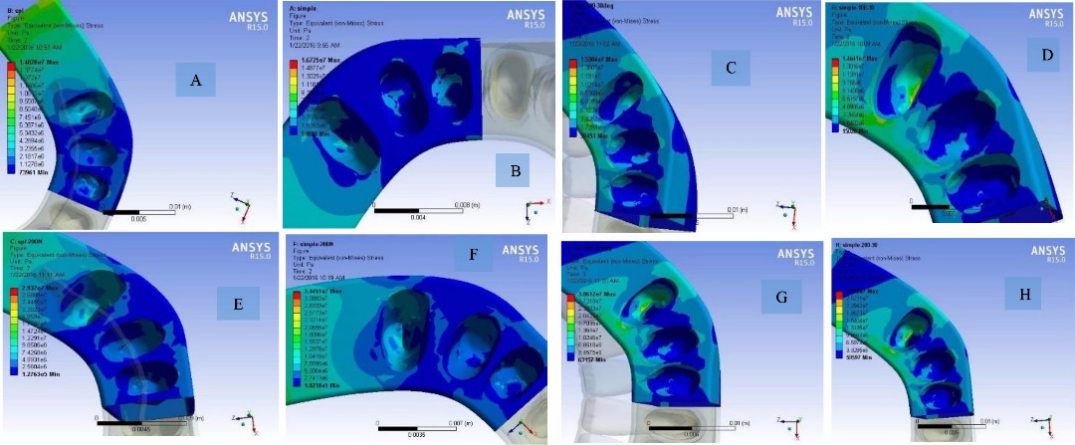
Maximum von Mises stress in the PDL of teeth in the splinted model under different loads and angles. (A) Maximum von Mises stress in the splinted model under 100-N load at 0° angle; (B) Maximum von Mises stress in the non-splinted model under 100-N load at 0° angle; (C) Maximum von Mises stress in the splinted model under 100-N load at 30° angle; (D) Maximum von Mises stress in the non-splinted model under 100-N load at 30° angle; (E) Maximum von Mises stress in the splinted model under 200-N load at 0° angle; (F) Maximum von Mises stress in the non-splinted model under 200-N load at 0° angle; (G) Maximum von Mises stress in the splinted model under 200-N load at 30° angle; (H) Maximum von Mises stress in the non-splinted model under 200-N load at 30° angle.

1. Maximum von Mises stress in the PDL of mandibular central incisor under 100-N load at 0° angle increased in the splinted model compared to the non-splinted model. Also, this stress in the PDL of mandibular lateral incisor and mandibular canine under 100-N load at 0° angle increased in the splinted model compared to the non-splinted model. The maximum von Mises stress in the cortical bone under 100-N load at 0° angle increased in the splinted model compared to the non-splinted model.

2. Maximum von Mises stress in the PDL of mandibular central incisor under 200-N load at 0° angle in the splinted model decreased compared to the non-splinted model. Also, this stress in the PDL of mandibular lateral incisor and canine under 200-N load and 0° angle increased in the splinted model compared to the non-splinted model. The maximum von Mises stress in the cortical bone under 200-N load and 0° angle increased in the splinted model compared to the non-splinted model.

3. Maximum von Mises stress in the PDL of mandibular lateral incisor under 100-N load at 30° increased in the splinted model compared to the non-splinted model. Also, this stress in the PDL of mandibular central incisor and canine teeth increased in the splinted model compared to the non-splinted model. This stress in the PDL of mandibular central incisor and canine teeth under 100-N load at 30° angle decreased in the splinted model compared to the non-splinted model. The maximum von Mises stress in the cortical bone under 100-N load at 30° angle increased in the splinted model compared to the non-splinted model.

4. Maximum von Mises stress in the PDL of mandibular lateral incisor under 200-N load at 30° angle increased in the splinted model compared to the non-splinted model. Also, this stress in the PDL of mandibular central incisor and canine teeth under 200-N load at 30° angle decreased in the splinted model compared to the non-splinted model. The maximum von Mises stress in the cortical bone under 200-N load at 30° angle decreased in the splinted model compared to the non-splinted model (Figure 2).

### Maximum shear stress in the two splinted and non-splinted models

Table 3 shows the corresponding data.

**Table 3:** Maximum von Mises stress (MPa) in the PDL of central and lateral incisors and canine teeth and the cortical bone surrounding them

**Load (N)**	**Non-splinted**				**Splinted**			
	**PDL of central** **incisors teeth**	**PDL of lateral incisors teeth**	**PDL of canine teeth**	**cortical bone**	**PDL of central** **incisors teeth**	**PDL of lateral incisors teeth**	**PDL of canine teeth**	**cortical bone**
**100 N load at 0°**	0.43	0.48	0.48	0.48	0.43	0.55	0.62	8.50
**200 N load at 0°**	0.85	0.96	0.96	0.96	0.83	1.03	1.23	12.29
**100 N load at 30°**	1.15	1.28	1.28	1.28	1.07	1.41	3.26	11.91
**200 N load at 30°**	2.21	2.59	2.59	2.59	2.13	2.81	6.52	23.82
**Load(N)**	**Non-splinted**				**Splinted**			
	**PDL of central** **incisors teeth**	**PDL of lateral incisors teeth**	**PDL of canine teeth**	**cortical bone**	**PDL of central** **incisors teeth**	**PDL of lateral incisors teeth**	**PDL of canine teeth**	**cortical bone**
**100 N load at 0°**	0.0086458	0.0099935	0.011712	0.00284	0.0089249	0.011507	0.012633	0.000621
**200 N load at 0°**	0.017292	0.019988	0.023423	0.001123	0.017239	0.021757	0.025448	0.0012
**100 N load at 30°**	0.02415	0.025857	0.067384	0.0010927	0.022508	0.029564	0.065731	0.0011347
**200 N load at 30°**	0.046502	0.052073	0.13439	0.001718	0.045001	0.059103	0.13141	0.00189

1. Maximum shear stress in the PDL of mandibular central incisor under 100-N load at 0° angle increased in the splinted model compared to the non-splinted model. Also, this stress in the PDL of mandibular lateral incisor and canine teeth under 100-N load at 0° angle in the splinted model increased compared to the non-splinted group. Maximum von Mises stress in the cortical bone under 100-N load at 0° angle increased in the splinted model compared to the non-splinted group.

2. Maximum shear stress in the PDL of mandibular central incisor under 200-N load at 0° angle decreased in the splinted model compared to the non-splinted model. Also, this stress in the PDL of mandibular lateral incisor and canine teeth under 200-N load at 0° angle increased by 8.11% in the splinted model compared to the non-splinted model. The maximum von Mises stress in the cortical bone under 200-N load at 0° angle increased in the splinted model compared to the non-splinted model.

3. Maximum shear stress in the PDL of mandibular lateral incisor under 100-N load at 30° angle increased in the splinted model compared to the non-splinted model. Also, this stress in the PDL of mandibular central incisor and canine teeth under 100-N load and 30° angle decreased in the splinted model compared to the non-splinted model. Maximum von Mises stress in the cortical bone under 100-N load at 30° angle in the splinted model decreased compared to the non-splinted model.

4. Maximum shear stress in the PDL of mandibular lateral incisor under 200-N load at 30° angle increased in the splinted model compared to the non-splinted model. Also, this stress in the PDL of mandibular central incisor and canine under 200-N load at 30° angle decreased in the splinted model compared to the non-splinted model. Maximum von Mises stress in the cortical bone under 200-N load at 30° angle decreased in the splinted model compared to the non-splinted model.

### Maximum von Mises strain in the two models

Table 3 shows the corresponding data.

1. Maximum von Mises strain in the PDL of mandibular central incisor under 100-N load at 0° angle increased in the splinted model compared to the non-splinted model. This strain in the PDL of mandibular lateral incisor and canine teeth under 100-N load and 0° angle increased in the splinted model compared to the non-splinted model. Maximum von Mises strain in the cortical bone under 100-N load and 0° angle decreased in the splinted model compared to the non-splinted model.

2. Maximum von Mises strain in the PDL of mandibular central incisor under 200-N load at 0° angle decreased in the splinted model compared to the non-splinted model. Also, this strain in the PDL of mandibular lateral incisor and canine teeth under 200-N load at 0° angle increased in the splinted model compared to non-splinted model. Maximum von Mises strain in the cortical bone under 200-N load at 0° angle increased in the splinted model compared to the non-splinted model.

3. Maximum von Mises strain in the PDL of mandibular lateral incisor under 100-N load at 30° angle increased in the splinted model compared to the non-splinted model. Also, this strain in the PDL of mandibular central incisor and canine teeth under 100-N load at 30° angle decreased in the splinted model compared to the non-splinted model. Maximum von Mises strain in the cortical bone under 100-N load at 30° angle increased in the splinted model compared to the non-splinted model.

4. Maximum von Mises strain in the PDL of mandibular lateral incisor under 200-N load at 30° angle increased in the splinted model compared to the non-splinted model. Also, this strain in the PDL of mandibular central incisor and canine teeth under 200-N load at 30° angle decreased in the splinted model compared to the non-splinted model. Maximum von Mises strain in the cortical bone under 200-N load and 30° angle increased in the splinted model compared to the non-splinted model.

## Discussion

Management of periodontally compromised teeth is a challenge in dentistry. Selection of an appropriate treatment plan customized for each patient is important since several factors might affect treatment planning for such cases. One important factor in the selection of a proper treatment plan is the biomechanical effects of each treatment plan on bone, which is important in the preservation of bone and prevention of bone loss. However, since it is difficult to clinically and paraclinically assess this factor, not many studies have been conducted in this field. Thus, this study aimed to biomechanically assess two common treatment plans for mobile teeth.^20-23^ In the current study, loads were applied at 30° angle, corresponding to the mean anterior guidance in patients with normal occlusion. During mastication, loads are applied to the anterior teeth at 30° angle. This angle in an individual with normal occlusion decreases during biting and may even reach 0° (parallel to the longitudinal axis of the tooth).^22^ Also, 100-N load is equal to the mean load applied to the mandibular anterior sextant during chewing and 200-N load is the maximum load applied during normal function.^23^

The current results indicated a significant reduction in maximum shear stress and maximum von Mises stress and strain in cortical bone around central and lateral incisors after splinting. In addition, maximum von Mises stress in the PDL of central and lateral incisors significantly decreased by splinting and a slight increase was noted in shear and von Mises stresses in the cortical bone around canine teeth. Shear stress and von Mises strain in the PDL before and after splinting did not exhibit significant changes. All these changes under 100-N load were more significant than 200-N load and showed greater difference under oblique loads compared to vertical loads. Moreover, stress distribution in the PDL and stress and strain in the cortical bone around roots under 30° loads were significantly different in the splinted model such that maximum stress concentration area changed from the crestal area in the non-splinted model to the apical region in the splinted model. Distribution of stress and strain in the PDL and cortical bone under vertical load did not show a significant difference. Chitumalla et al^24^ assessed a model including a four-unit crown and bridge with mandibular first and second premolar abutments. Both teeth had lost one-third of their periodontal support. They applied a 200-N vertical load to the occlusal surface (parallel to the long axis of the teeth) and reported that maximum stress in cortical bone around teeth was 137 and 160 MPa for the molar and premolar teeth; these values are much higher than the values reported in the splinted model in the current study under 200-N vertical load. The greatest numerical difference was due to the method of load application and its effects such that 200-N load was applied to separate points on the designed crowns. This means that the sum of loads applied to the four-unit crown and bridge was 800-N. This stress was distributed in the tissue supporting the two splinted abutments. However, in the current study, 200-N load was applied to six anterior teeth in the form of a sheet. It means that stress due to the application of 200-N load is distributed in the tissues supporting the splinted six anterior teeth. Thus, in their study, compared to ours, higher load was distributed in a smaller surface and higher concentration of stress was expected. Also, the posterior teeth were evaluated by Chitumalla et al,^24^ and two pontic units were present between the two abutments while anterior teeth with no bridge were evaluated in our study, which also justifies the difference in stress concentration.

In a study by Kurgan et al,^25^ two models with 40% bone level with and without FRC splinting, which were highly similar to the models in our study, were evaluated. They reported maximum von Mises stress under 100-N vertical load to be 145 and 130 MPa in non-splinted and FRC splinted models, respectively; these values were considerably higher than the values reported in our study. Part of this difference might be attributed to the higher bone level in our study since higher bone level results in greater contact area of root with the supporting structures and thus, the applied load is distributed in a larger surface. This can decrease maximum von Mises stress value in bone around roots. Also, this numerical difference between our study, and that of Kurgan et al,^25^ might be due to the absence of modeling the PDL structure around roots since PDL with its low modulus of elasticity absorbs parts of stress and results in better stress distribution in the underlying bone. This decreases the maximum von Mises stress in cortical bone. On the other hand, Kurgan et al^25^ applied load to a point on the tooth surface, which is different from the clinical situation and results in the application of up to 600-N load to mandibular anterior teeth, which is much higher than the load applied to teeth in the current study. This value is also several times higher than the load applied by the muscles of mastication of humans to teeth in the anterior region. In other words, clinical setting was not well simulated in their study and this is the main reason explaining the difference in values obtained in their study and ours.^25^ Kumbuloglu et al^26^ showed improved periodontal condition of patients with bone loss in the anterior mandible, which was expected considering our current findings. However, since they did not have a non-splinted control group in their study, comparison of splinted and non-splinted groups was not performed in their study. Thus, their study cannot be accurately compared with ours. Wakabayashi et al, in 2012, assessed splinted and non-splinted models of two premolar teeth, one with 5 mm and the other with 2 mm of bone loss, which were subjected to 18.6-44.8 and 292-N loads, similar to our study. However, due to the assessment of different factors in the two studies, numerical comparison of the models in the two studies was not possible. They assessed compressive strain in bone and tensile stress in roots while in the current study, the von Mises strain in bone, which is the result of strain in different directions, was assessed. Moreover, we assessed different forms of stress in the PDL and bone around teeth and not in the root structure. They compared the two models and showed a reduction in maximum stress and strain in root and alveolar bone around teeth with 5 mm of bone loss and an increase in stress and strain in teeth with better periodontal support (2 mm of bone loss) following splinting of the two teeth; these results were in line with our findings regarding comparison of splinted and non-splinted models (indicating improvements in the conditions of central and lateral incisors and worsening of the condition of canine teeth).^5^

## Conclusion

Our study showed that the tooth with the best bone support (right and left canine) experienced greater stress after splinting. Prior to splinting of teeth in the anterior mandible, adequate hone support of canine teeth must be ensured to increase the longevity of the system. Splinting of highly mobile teeth to teeth with adequate bone support can increase the stress and compromise biomechanical health. Further studies on other treatment plans for highly mobile teeth with periodontal disease by use of 3D FEA are required to compare splinting with other available treatment plans.

## Competing interests

The authors declare no competing interests with regards to the authorship and/or publication of this article.

## Ethics approval

Not applicable.
